# Occurrence and toxigenic potential of *Aspergillus* section *Flavi* on wheat and sorghum silages in Uruguay

**DOI:** 10.1080/21501203.2020.1752321

**Published:** 2020-04-27

**Authors:** Agustina del Palacio, Dinorah Pan

**Affiliations:** Laboratorio de Micología, Facultad de Ciencias, Facultad de Ingeniería, UdelaR, Montevideo, Uruguay

**Keywords:** Silage, aflatoxin, cyclopiazonic acid, *Aspergillus flavus*

## Abstract

Species belonging to *Aspergillus* section *Flavi* occur naturally in crops and can cause food spoilage and/or toxin production. The aim of this study was to determine the occurrence and diversity of the species of *Aspergillus* section *Flavi* found in wheat and sorghum at harvest time and during silage storage, and to evaluate the toxigenic potential of the isolates to determine the contamination risk of mycotoxins in grains. Strains from *Aspergillus flavus* and *Aspergillus parasiticus* were found based on multi-gene phylogenetic analyses. This is the first report on the presence of *A. parasiticus* in wheat from Uruguay. Of the 80 isolates *Aspergillus* section *Flavi*, 30% produced aflatoxins (AFs), mainly type B1, and 25% produced cyclopiazonic acid (CPA). Within the isolates from wheat samples, 35% were AFs producers and 27.5% were CPA producers. Among the *Aspergillus* section *Flavi* isolates from sorghum, 25% were AFs producers while 22.5% were CPA producers. This work contributes to the knowledge of the species in crops and helps define appropriate strategies for the prevention and control of contamination with AFs and CPA by *Aspergillus* section *Flavi* fungi.

## Introduction

Wheat is one of the most widely grown crops in the world and is extensively used for human consumption due to its high nutritional value (Hawkesford et al. 2013). Although it is one of the most important cultivated cereals in Uruguay for human consumption, when harvest yield is low it is also usual to be ensiled for animal feeding (OPYPA 2018).

Sorghum grains are used as raw material for poultry, swine and bovine feeds, but are also destined for human use in different parts of the world (Oniang’o et al. 2003; Pena et al. 2019). In Uruguay, even though pastures constitute the major source for dairy cattle feeding, sorghum silage is also used in a ratio of 125 grams per litre of milk produced (DIEA 2013). Since 2015 in Uruguay sorghum has also been used to produce bioethanol as well as its sub-product, dried distillers grains with soluble (DDGS), is used for animal feeding (Methol 2018).

Silage is a forage preservation method that allows its storage during long periods of time maintaining its nutritional value comparable to fresh pastures. Grains stored under silo conditions are vulnerable to contamination by spoilage moulds and mycotoxins because they are excellent substrates for fungal growth, thus representing an important problem for human and animal health (Driehuis 2013). Due to contamination the costs of food production increase because of testing needs. Also, contaminated loads lower the prices and there may be potential lawsuits from consumers. In addition, there may be a decrease in livestock performance (Ferrero et al. 2019).

Aflatoxins (AFs) are toxic secondary metabolites produced by several species of *Aspergillus* section *Flavi* that frequently contaminate important staples such as maize, peanuts and nuts (Sweeney and Dobson 1998). They are carcinogenic, teratogenic, immunosuppressive and genotoxic compounds that have been classified by the International Agency for Research on Cancer (IARC) as group 1 carcinogens (IARC 2012). Four compounds are commonly produced in foods, aflatoxin B1 (AFB1), aflatoxin B2 (AFB2), aflatoxin G1 (AFG1) and aflatoxin G2 (AFG2). For example in milk, other bio-transformed aflatoxins may occur, such as aflatoxin M1 (AFM1) and aflatoxin M2 (AFM2) (Cole and Cox 1981). Cyclopiazonic acid (CPA) is an indole-tetramic acid toxic to a variety of animals and has also been implicated in human poisoning (Luk et al. 1977; Rao and Husain 1985; Antony et al. 2003). Toxic effects include: liver degeneration and necrosis, myocardial lesions, decreased weight gain, vomiting, kidney lesions, pancreas, spleen and several neurotoxic symptoms (Kuilman-Wahls et al. 2002; Duran et al. 2007).

Aflatoxins are highly regulated in human and animal food in more than 100 countries throughout the world (Wu 2015). Uruguay has established limits for total AFs in infant food (3 µg/kg) and in food products for human consumption (20 µg/kg) as well as for AFM1 in milk and derivatives (0.5 µg/kg) (RBU 1994). However, there is no regulation for AFs in feed and feed ingredients in Uruguay. At present, there are no regulations for CPA in foods and feeds in the world.

The most important AFs producers from a public health point of view are *Aspergillus flavus* and *Aspergillus parasiticus*, both belonging to *Aspergillus* section *Flavi*. However, in the last decade, section *Flavi* has been studied in depth using molecular tools and several new species have been identified. This section currently comprises 35 different species of which 20 are aflatoxigenic (Carvajal-Campos et al. 2017; Singh et al. 2018; Frisvad et al. 2019). These species can be distinguished by subtle morphologic characteristics, gene sequences and by their ability to produce different mycotoxins (Bailly et al. 2018). Two species only produce AFB1 and AFB2 (*A. pseudotamarii* and *A. togoensis*), and 15 species are able to produce AFB1, AFB2, AFG1 and AFG2: *A. aflatoxiformans, A. austwickii, A. cerealis, A. arachidicola, A. minisclerotigenes, A. mottae, A. luteovirescens, A. nomius, A. novoparasiticus, A. parasiticus, A. pseudocaelatus, A. pseudonomius, A. sergii, A. texensis* and *A. transmontanensis* (Singh et al. 2018; Frisvad et al. 2019). *Aspergillus flavus* isolates can produce AFB1 and AFB2 or CPA, or both, or neither. Generally, AFGs are not produced by this species, although some studies have reported AFG production by isolates identified as *A. flavus* (Baranyi et al. 2015; Camiletti et al. 2017; Okoth et al. 2018; Saldan et al. 2018; Frisvad et al. 2019). *Aspergillus oryzae* and *A. sojae* appear to be the domesticated forms of the aflatoxigenic species *A. flavus* and *A. parasiticus*, respectively, and they are used extensively in food and biotechnology industries (Houbraken et al. 2014).

Another distinctive characteristic of *Aspergillus flavus* isolates is the production of sclerotia, structures that serve as infective propagules in soil. Some isolates, called S strains, produce abundant small sclerotia (<400 µm in diameter) while L strains produce fewer but larger sclerotia (>400 µm in diameter) (Cotty 1989). The L strains produce variable quantities of AFs, and isolates can either be atoxigenic or produce moderate to high levels of AFs; however, the S strains of *A. flavus* are known to consistently produce higher concentrations of AF (Cotty 1989; Cotty and Cardwell 1999; Chang et al. 2001; Novas and Cabral 2002; Probst et al. 2010). Despite this, the correlation between sclerotial size and AF production ability has not been observed by other authors (Razzaghi-Abyaneh et al. 2006; Giorni et al. 2007).

Although several species of section *Flavi* can produce high levels of AFs in crops when are present in a conducive environment, genotypes vary in their potential to produce AFs and their relative importance as etiologic agents that may vary from one region to another (Cotty et al. 2008). Considering this, and due to the little information in Uruguay about contamination with species that produce aflatoxin in wheat and sorghum silages, the aim of this study was to: i) determine the occurrence and diversity of the species of *Aspergillus* section *Flavi* present in wheat and sorghum silages and ii) evaluate the toxigenic potential of the isolates in order to determine the risk of contamination with mycotoxins in these grains and in this kind of storage. This knowledge has important practical implications for the development of appropriate storage strategy for each crop in order to reduce AFs contamination. Also, the knowledge about the presence of aflatoxigenic species and their aflatoxigenic potential is crucial to monitoring introduction of new species of section *Flavi* responsible of toxins production.

## Materials and methods

### Silage samples and fungal isolation

The isolates of *Aspergillus* section *Flavi* used in this study (40 isolates from wheat and 40 isolates from sorghum) were collected from samples of wheat (variety Baguette) and sorghum (variety Flash 10) harvested from cultivated lands of farms located at the south-west region of Uruguay, mechanically chopped and enclosed in a polystyrene 250 µm bag thick. The silo bags were 60 m long, 2.5 m diameter and 1.7 m height; they were filled with about 180 tons of cereals and then hermetically sealed. A total of 40 samples of wheat (4 from freshly harvested grains and 36 from stored grain) were analysed from November 2009 at harvest time and at 60, 90 and 120 days of ensiling (Give more informations about storage conditions T° & aw). A total of 50 samples of sorghum (5 from freshly harvested grains and 45 from stored grain) were analysed from May 2011 at harvest time and at 30, 90 and 180 days of ensiling. Table 1 shows the physical properties of the silages. Sampling was performed manually through the silos in transects at three levels (upper, middle and lower). From the cut edge, three points from each level and from three equidistant points along the silo were sampled at 50 cm horizontal depth. At each time, 1 kg samples from each point were collected, homogenised and quartered to obtain 500 g sub-samples for analysis.

One hundred wheat grain particles and 100 sorghum grain particles from each sample were placed in 10 Petri dishes (10 grains particle per plate) containing potato dextrose agar (PDA) and incubated at 25°C under a 12 hs white/12 hs black fluorescent light photoperiod for 7 days. The colonies presumably belonging to *Aspergillus* section *Flavi* based on macro and micromorphological characters such as olive green/yellow conidia, conidial heads mainly radiate with usually quite rough stipes and conidia globose to ellipsoidal, were transferred to Petri dishes with PDA for the subsequent identification to species level.

### Morphological characterisation of the isolates

Following conventional mycological methods the identification was performed according to the taxonomic keys and guides available for the Aspergillus genus (Pitt and Hocking 1997; Klich 2002). For this, each isolate was inoculated in three points on plates containing Czapek Yeast Extract Agar (CYA), at 25°C and 37°C; Malt Extract Agar (MEA), Czapek Dox (CZ) and Czapek Yeast Extract with 20% Sucrose Agar (CY20 S) at 25°C. All dishes were incubated for 7 days and colony diameter was then measured and analysed for colony colour, head seriation and conidia morphology.

### Sclerotia production

Each isolate was inoculated into Petri dishes containing CZ and 5/2 agar and incubated at 30°C in the dark for 15 days (Mauro et al. 2013; Alaniz Zanon et al. 2016). Sclerotia were obtained according to Novas and Cabral (2002) by scraping the surface of the plate over a N°2 Whatman filter paper during irrigation with water containing Tween 20 (100 µl/l), followed by rinsing with running tap water. Sclerotia were further cleaned in a beaker with repeated rinses and decanting, and later were air-dried. To assess diameters, sclerotia were spread out on the gridline plate and measurements of 20 sclerotia were recorded. Isolates of *A. flavus* were identified as “S” (< 400 µm in diameter) or “L” (> 400 µm in diameter) according to Cotty (1989).

### Genetic identification of the isolates

The parts of the ß-tubulin (*BenA*) using primers bT2a 5ʹ-GGTAACCAAATCGGTGCTGCTTTC-3ʹ and Bt2b 5ʹ-ACCCTCAGTGTAGTGACCCTTGGC-3ʹ and calmodulin (*CaM*) genes using primers CMD5 5′-CCGAGTACAAGGAGGCCTTC-3′ and CF4 5′-TTTYTGCATCATRAGYTGGAC-3ʹ were amplified for the identification of 80 *Aspergillus* spp. isolates (Varga et al. 2011). Genomic DNA was extracted using the cetyltrimethylammonium bromide (CTAB) method described by Lee and Taylor (1990). Each PCR reaction contained 20–30 ng of genomic DNA, 10X PCR buffer, 100 mM each of dATP, dCTP, dGTP, and dTTP, 100 nM each of forward and reverse primers and 0,5 U Taq DNA polymerase (Fermentas International Inc., Canada) in a total volume of 50 µL. PCR reactions were carried out in a GeneAmp PCR system 9700 thermocycler (Perkin-Elmer, USA) using the following cycling protocol: an initial denaturation step of 95°C for 2 min; 35 cycles of 95°C for 30 s, 54°C (*BenA*) or 58°C (*CaM*) 30 s, 72°C for 30 s; final extension of 72°C for 10 min. Then, the purified PCR products were sequenced by Macrogen Inc., Korea. The consensus sequences of the PCR products were obtained using the SeqMan software (Lasergene, Madison, WI) and compared with the sequences of closely related species in GenBank database by using the Basic Local Alignment Search Tool (BLAST). The sequences obtained herein were aligned with the type strains sequences from all *Aspergillus* section *Flavi* species. Phylogenetic trees for gene combined alignment were inferred by using the Maximum Parsimony method with partial deletion and Tree-Bisection-Regrafting (TBR) algorithm (Nei and Kumar 2000). *Aspergillus muricatus* was used as out group. The analyses were conducted in the software MEGA version X (Kumar et al. 2018) with 1000 bootstrap replicates for assessing node confidences.

### Toxigenic capacity of fungal isolates

#### Aflatoxin production

Aflatoxins analyses were performed using the methodology described by Bragulat et al. (2001). The strains were incubated in Petri dishes containing yeast extract sucrose (YES agar, Katsurayama et al. 2018) at 25ºC for 14 days in the dark. Then, 3 plugs from each Petri dish were transferred to an Eppendorf tube and 1 ml of methanol was added. After 1 hour, the solution was filtered with a Millipore filter (Ø 0.22 mm), an aliquot (200 µl) was derivatised with 700 µl triﬂuoroacetic acid: acetic acid: water (20:10:70, v/v/v). The derivatised solution was analysed using a reverse phase HPLC consisting of a Shimadzu LC-10ADvp pump, a RF-10Axl fluorescence detector (Shimadzu; excitation and emission wavelength of 360 nm and 440 nm, respectively), and a C18 reversed-phase column (150 mm x 4.6 mm i.d., 5 µm particle size; Nucleodur®, Macherey-Nagel, Düren, Germany) connected to a pre-column Security Guard (8 mm x 4 mm i.d., 5 µm particle size; Nucleodur®, Macherey-Nagel, Düren, Germany). The mobile phase was water: methanol: acetonitrile (4:1:1, v/v/v) at a flow rate of 1.5 ml min-1. The injection volume was 20 µl. Aflatoxins production was measured in ng g-1 of culture medium. The limit of detection was 1 ng g-1 of AFB1 and AFG1, and 0.8 ng g-1 of AFB2 and AFG2.

#### Cyclopiazonic acid production

The isolates were inoculated at a single central point on Petri dishes (Ø 6 cm) containing CZ and incubated at 25°C for 14 days in the dark. Three agar plugs were removed from different points of the colony and extracted with 1 ml of methanol. The mixture was centrifuged at 12,000 rpm for 13 min and the supernatant was evaporated to dryness. The residue was re-dissolved in methanol for thin layer chromatography (TLC) on silica gel 60 plates (Macherey-Nagel, Germany). The plate was immersed in oxalic acid in ethanol 2% for 10 min, heated at 80°C for 1 h and cooled. Benzene: acetic acid: methanol (90:5:7) was used as developing solvent. Cyclopiazonic acid was visualised after treatment of the plates with p-dimethilaminobenzaldehido 1% on 75 ml of ethanol and 25 ml of chloride acid, with subsequent development of blue spots (Horn and Dorner 1999). The detection limit was 1.5 ng g-1.

## Results

### Aspergillus *section* Flavi *across storage time*

**Figure 1.**
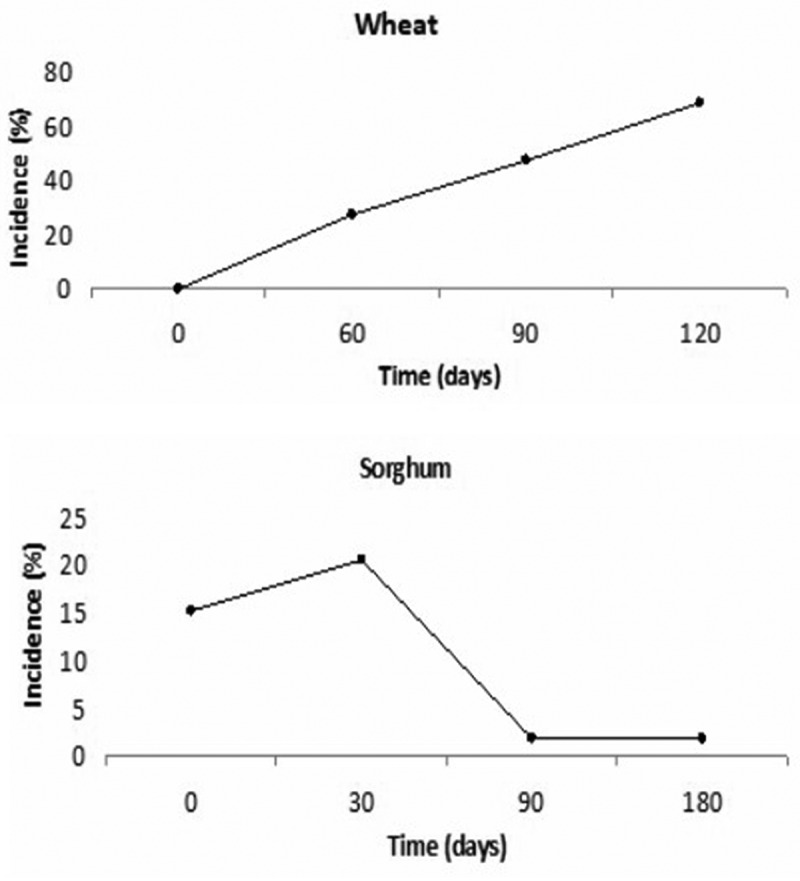
Incidence(%) of *Aspergillus* section *Flavi* throughout storage time in both silages.

In wheat grain, *Aspergillus* section *Flavi* was present with an incidence of 4% at harvest and during the different storage stages, with incidences of 27.5% at 60 days and 47% at 90 days of silage. After 120 days of storage, the incidence in the samples reached 67% (Figure 1). Also, the presence of *Aspergillus* section *Flavi* exhibited a positive correlation with time of storage (r = 0.99, *p* < 0.05). On the other hand, *Aspergillus* section *Flavi* showed significant differences during the sampling time (*p* < 0.05) in sorghum grain. The highest incidence was at 30 days of storage (21%), after this it became significantly less frequent (2%) (*p* < 0.05) (Figure 1). No correlation was found between *Aspergillus* section *Flavi* and time of storage (*p* > 0.05).

On the basis of morphological characteristics all isolates showed typical morphological features of olive green colonies with conidial heads mainly radiate and uniseriate with smooth to finely rough globose conidia and usually quite rough stipes. Based on these morphological characteristics and the measures of growth in the different culture media used, all strains were identified as *Aspergillus flavus* (Supplementary Table S1 and S2).

**Figure 2.**
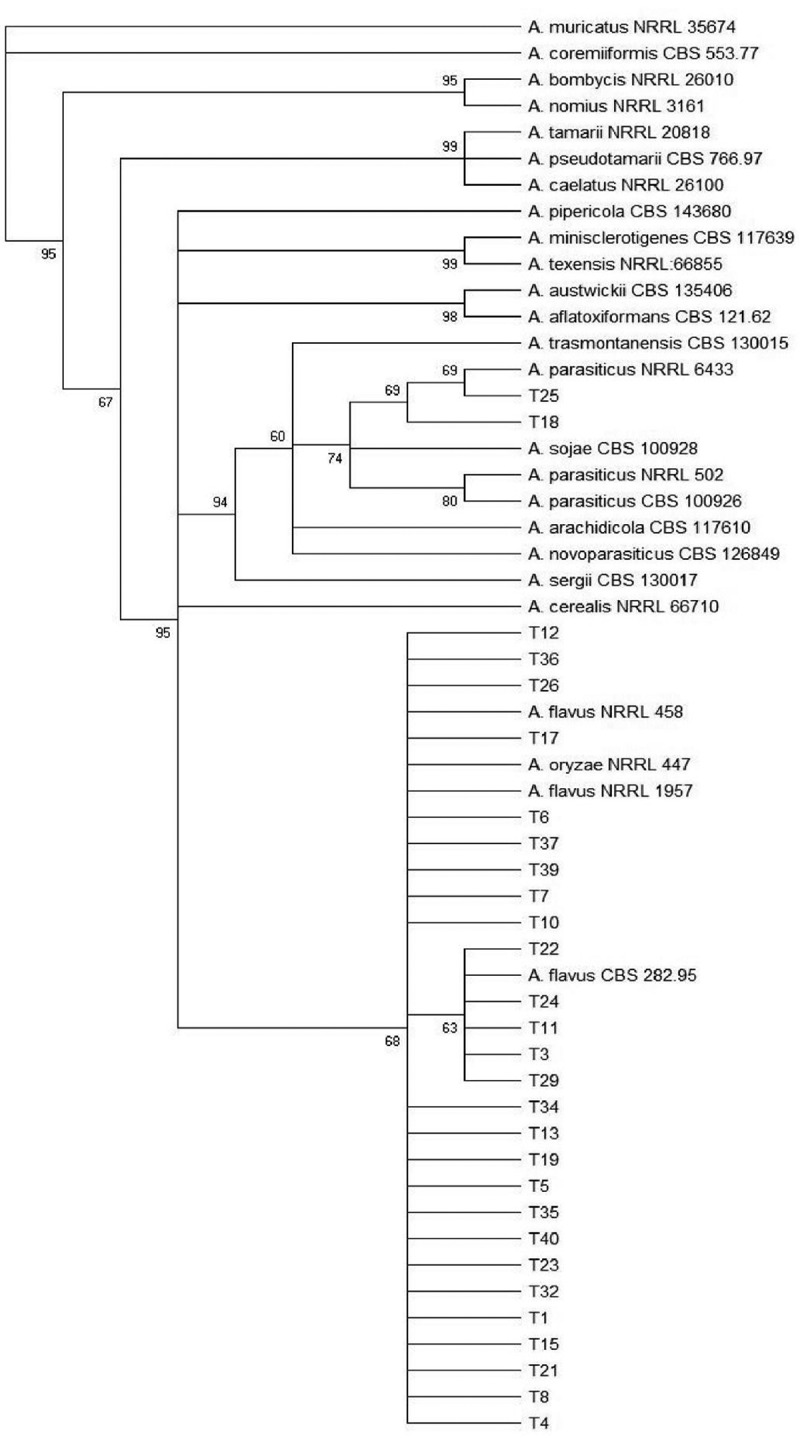
Maximun parsimony (MP) tree from the combined alignment of calmodulin and b-tubulin gene sequences data from *Aspergillus* section *Flavi* isolates from wheat. The bootstrap values (≥60%) are indicated above each node.

**Figure 3.**
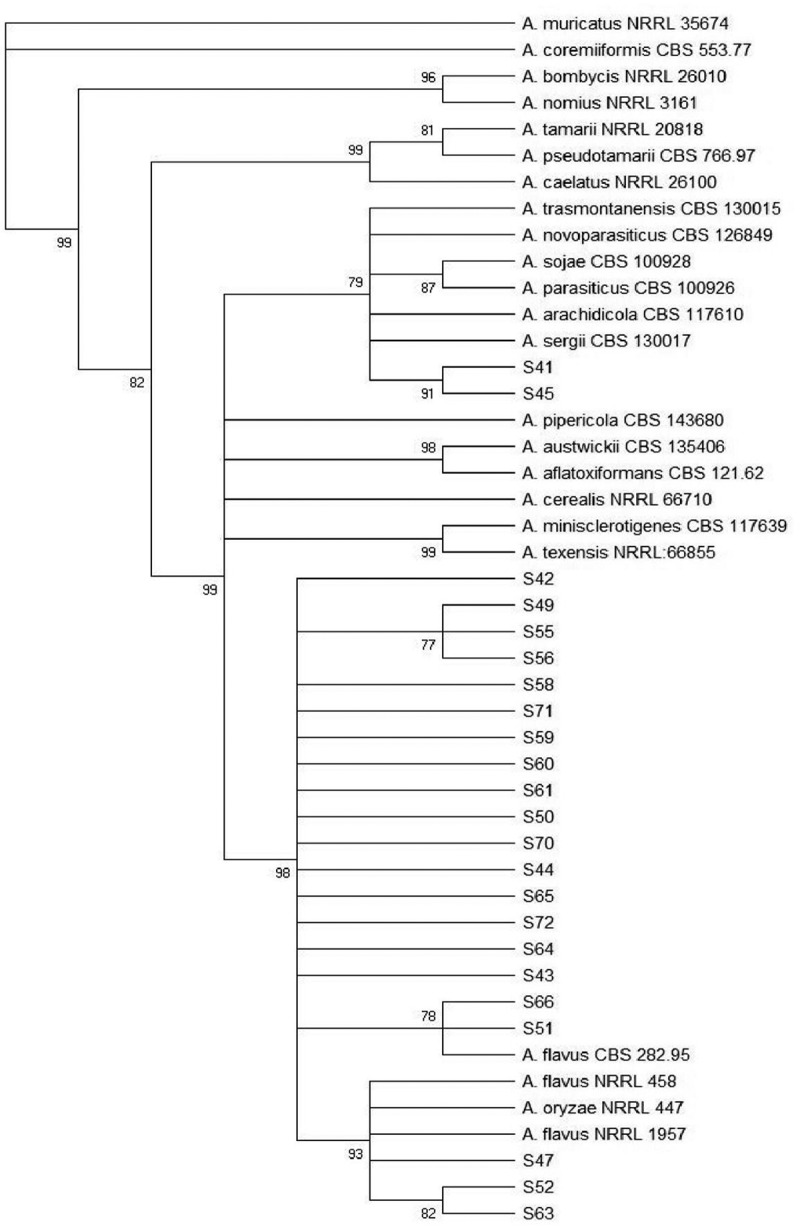
Maximun parsimony (MP) tree from the combined alignment of calmodulin and b-tubulin gene sequences data from *Aspergillus section Flavi* isolates from sorghum. The bootstrap values (≥70%) are indicated above each node.

**Figure 4.**
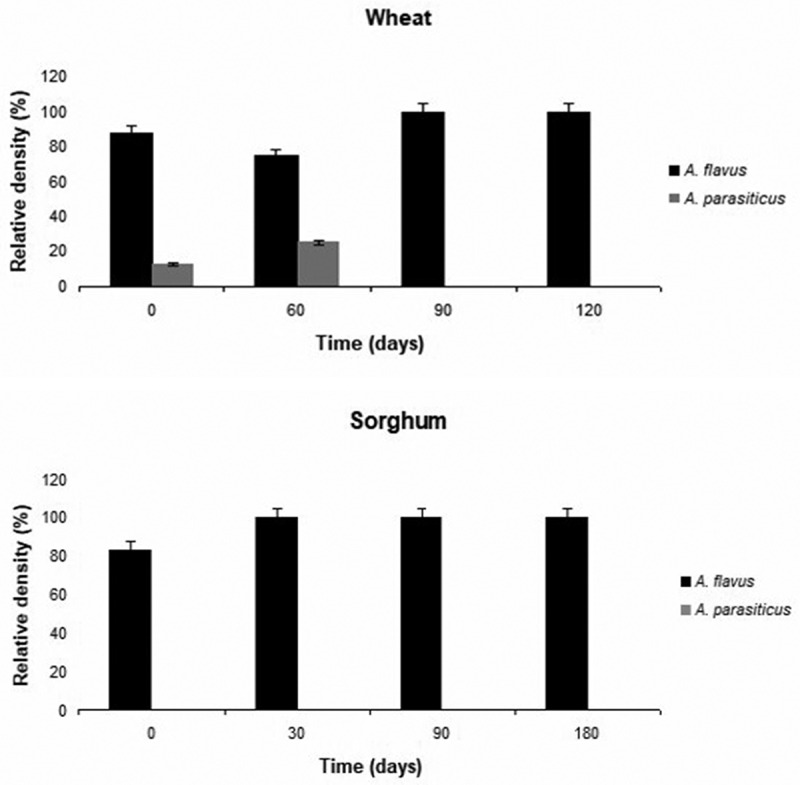
Relative density (%) of *Aspergillus Flavi* and *A.* *parasiticus* throughout storage time in both silages. The numbers 0, 30, 60, 90, 120 and 180 reperesent the storage times in days in silos.

According to the identification using morphological markers and a phylogenetic study using combined data sets of genomic sequences of two genes (*BenA* and *CaM*), the isolates belonging to section *Flavi* were *A. flavus* (n = 76; 95%) and *A. parasiticus* (n = 2; 2,5%). In addition, two sorghum strains could not be identified to species level, but belong to *A. flavus* clade (n = 2; 2,5%). The phylogenetic trees of *Aspergillus* section *Flavi* of wheat and sorghum isolates are shown in Figures 2 and 3. Figure 4 shows the relative density of these species according to time of sampling in wheat and sorghum. In both grains, *A. flavus* was the predominant species over storage time and *A. parasiticus* was absent in wheat grains after 90 days of storage.

### Sclerotia production

Of the isolates of *Aspergillus* section *Flavi*, 83.7% (n = 67) formed sclerotia under culture conditions. Of these isolates, 87.5% (n = 35) belonged to wheat and 80% (n = 32) to sorghum showing significant differences between both (*p* < 0.05). Among the isolates of wheat, 89.5% (n = 34) of *A. flavus* were strains that produced sclerotia and just one isolate was of *A. parasiticus*. While among the isolates of sorghum, 81.6% (n = 31) of *A. flavus* produced sclerotia. All *A. flavus* isolates of wheat that were sclerotia producers were classified as L strains, whereas in sorghum 29 isolates of *A. flavus* (93.5%) were classified as type-L morphotype and 2 isolates (6.5%) as type-S morphotype (S43 and S44).

### Mycotoxin production

Of the 80 isolates from *Aspergillus* section *Flavi*, 30% (n = 24) produced AFs in culture and 25% (n = 20) produced CPA. Within the isolates from wheat samples, 35% (n = 14) were AFs producers and 27.5% (n = 11) were CPA producers. Among the *Aspergillus* section *Flavi* isolates from sorghum, 25% (n = 10) were aflatoxigenic while 22.5% (n = 9) were CPA producers. Table 2 shows the toxigenic capacity of *Aspergillus* section *Flavi* isolates from wheat and sorghum samples. No statistically significant differences were observed when comparing the mycotoxigenic profile between wheat and sorghum isolates (*p* < 0.05). Overall, more atoxigenic strains were detected throughout storage time in both silages. In wheat silages, isolates that produced AFB1 were predominant and significantly different from the other AFs profile (*p* < 0.05), whilst those that produced AFB1 and AFB2 were predominant and significantly different from the other AFs profile in sorghum silages (*p < *0.05).

**Table 2.** Mycotoxigenic profile of *Aspergillus* section *Flavi* isolates in both silages.

	Isolates of *Aspergillus* section *Flavi* (%)					
	AFB	AFG	AF B + G	CPA	AF + CPA	atoxigenic
Wheat (n = 40)	7.5	5.0	7.5	12.5	15.0	52.5
Sorghum (n = 40)	5.0	5.0	5.0	12.5	10.0	62.5

Of the 76 strains from *A. flavus*, 15.6% (n = 12) produced AFB, 5.3% (n = 4) produced AFG, 6.6% (n = 5) produced both AFB and AFG, 11.8% (n = 9) produced CPA and 13.2% (n = 10) produced AFs and CPA. *Aspergillus parasiticus* strains (n = 2) were able to produce both AFB and AFG.

## Discussion

Most of the studies about *Aspergillus* section *Flavi* are focused on corn silages (Alonso et al. 2013) and in a minor extent on wheat and sorghum silages (Del Palacio et al. 2016a, 2016b; Divakara et al. 2014; Keller et al. 2012; Riba et al. 2010; Yuan et al. 2018). Despite this, there is now available information about the composition of communities of *Aspergillus* section *Flavi* in wheat and sorghum grains when it comes to storage under silo bag conditions. Therefore, this is the first study to perform morphological, molecular and chemical characterisation of Uruguayan isolates of *Aspergillus* section *Flavi* associated with wheat and sorghum from the field to storage.

For both substrates analysed, the infection in the field was lower than the one found during storage. In wheat samples the positive correlation between incidence and storage time suggests silo conditions to be ineffective against *Aspergillus* section *Flavi* infection in this substrate unlike what was observed in sorghum. This may be due to the high pH levels of the silo that ranged from 6.4 to 6.7 (Table 1). Grain silage storage is based on the chemical processes that occur in vegetable tissues when they are under anaerobic conditions and in the presence of lactic acid bacteria. This leads to a decrease in pH value (4–4.5) and inhibits several spoilage microorganisms. Fungal growth generally occurs when the silo is not well packed and pH values ranged over 6 (Alonso et al. 2013; Ferrero et al. 2019).

On the other hand, ensiled sorghum for periods longer than 30 days, reduce the presence of species of *Aspergillus* section *Flavi* when a good management of silo practices are followed. This indicates that stored sorghum grains could be less frequently infected by *Aspergillus* section *Flavi* than other grains such as wheat and corn, and that sorghum could be a more adequate substrate to be used in silage.

This is the first report of the presence of *A. parasiticus* in wheat grains of Uruguay. Within the section *Flavi*, only *A. flavus* and *A. parasiticus* were identified. *Aspergillus flavus* was found at high relative density before and during the storage time in both silages, showing that this species is well adapted to storage conditions. Similar results were obtained by other authors in corn silages (El-Shanawany et al. 2005; Keller et al. 2013; Ferrero et al. 2019). However, this result differs from that found by Yuan et al. (2018) in which toxigenic *Aspergillus* species were at very low levels before and during wheat grain storage. On the other hand, *A. parasiticus* was present in field and was absent after 90 days of storage in wheat grains. This is important considering that this species can produce AFB and AFG and most of the strains have the capacity to produce both (Varga et al. 2011). More ecophysiological studies need to be done on these species to understand the prevalence of *A. flavus* and the absence of *A. parasiticus* under silage conditions.

Sclerotia are survival structures resistant to adverse environmental conditions. In addition, sclerotia of section *Flavi* germinate sporogenically in soil by producing aerial conidiophores, which represent a source of primary inoculum in crops (Horn et al. 2014). In this study, *Aspergillus flavus* population found in both grains consisted mainly of strains that produced sclerotia, being type-L morphotype the most prevalent. This suggests that strains from Uruguay could remain as infective propagules in soil or grain for long periods of time increasing the risk of mycotoxins in grains. Type-L strains (> 400 µm) were more abundant than S strains as it was reported in other studies (Pildain et al. 2004; Giorni et al. 2007; Atehnkeng et al. 2008; Donner et al. 2009; Astoreca et al. 2011; Kachapulula et al. 2017). Type S strains are frequently found in relatively low rainfall and high temperatures regions (Bigelow et al. 2000; Cardwell and Cotty 2002; Singh et al. 2018). This may explain the low frequency of type-S strains found in our country.

Various studies refer to a higher AFs production by isolates with small sclerotia (Garber and Cotty 1997; Chang et al. 2001; Novas and Cabral 2002; Barros et al. 2006) whereas others, report no correlation between sclerotial size and AFs production (Razzaghi-Abyaneh et al. 2006; Giorni et al. 2007; Astoreca et al. 2011). In this work, although one of the S strains produced the highest levels of AFB1 (>15.000 µg/kg), the other strain was not able to produce AFs. On the other hand, most of the type-L strains did not produce AFs. The aforementioned relationship cannot be confirmed nor rejected and therefore a greater number of S strains would need to be analysed.

The 70% of atoxigenic strains of *A. flavus* found in the present study was relatively high, of which 55.3% corresponded to wheat and 65.8% to sorghum. These results are in agreement with those found by da Silva et al. (2004); Katsurayama and Taniwaki (2017); Mauro et al. (2013); Razzaghi-Abyaneh et al. (2006) and Vaamonde et al. (2003) in wheat, sorghum, maize, rice and soybean. The high frequencies of atoxigenic strains of *A. flavus* might be associated with the dominance of type-L morphotype found in the current study which is known to have a high incidence of atoxigenic strains (Mauro et al. 2013). On the other hand, these native atoxigenic strains could be organisms of interest to develop bio-control strategies for reducing AFs contamination in grains cultivated in the region. Highly competitive atoxigenic strains might be applied to agricultural fields as biological control agents (Sarrocco and Vannacci 2018).

In the present study, 30% of the isolates were able to produce AFs and 25% CPA, either as a sole toxin or both toxins simultaneously. In addition, most of the isolates were able to produce only AFs, being the mycotoxin production profile predominant in both grains. Despite this, the toxigenic profile of the *Aspergillus* strains observed in this work was highly variable suggesting possible high levels of genetic recombination among members of these species (Mamo et al. 2018). Olarte et al. (2012) showed that there is a high heritability, genetic variability and recombination at AFs gene cluster in *A. flavus* strains, and that sexual reproduction leads to spontaneous recombination between different populations. Moreover, due to the association of sclerotia with the sexual stage of *A. flavus* and the high percentage of strains obtained able to produce sclerotia, it would be expected that our population present a high genetic recombination (Horn et al. 2009). This could explain why some of the *A. flavus* strains obtained here produced AFB and AFG. It is known that *A. flavus* can produce AFB1 and AFB2 but not AFG1 and AFG2. However, recently reports have shown that *A. flavus* can produce AFs of the G type (Okoth et al. 2018; Frisvad et al. 2019).

Strains of *Aspergillus* section *Flavi* able to produce AFB, AFG and CPA were found. This mycotoxin proﬁle along with the production of small sclerotia are the distinctive characteristics of the recently described species: *A. aflatoxiformans, A. austwickii, A. cerealis, A. minisclerotigenes, A. mottae, A. pipericola, A. pseudocaelatus, A. sergii* and *A. texensis*, while *A. flavus* and *A. pseudotamarii* produce only AFB in addition to CPA. Despite this, the strain found here (S43) which had those characteristics was phylogenetically associated with *A. flavus*. Further investigation should be done with strains 41 and 45 isolated from sorghum that could not be identificated to species level and were distinct from *A. flavus*.

Our study also demonstrated the possible co-contamination of silages with AFs and CPA. Besides AFs, *A. ﬂavus* often produces CPA, an indole-tetramic acid that is toxic to a variety of animals and humans (Chang et al. 2009). In the present study, 29% (n = 11) of the *A. flavus* isolates from wheat and 21% (n = 8) from sorghum were CPA producers. Such co-production (AFs + CPA) may increase the toxicological risks since possible toxic synergies between these two mycotoxins could be important to animal health and potentially to human food safety (Maragos et al. 2017).

Mycotoxin production in *A. flavus* is highly variable and depends on several factors such as genotype, substrate, geographic origin, climate conditions, and agronomic practices. In our work, no significant differences were observed in the toxigenic profile of the isolates during storage time. However, strains that produced AFB2 were predominant in sorghum but not in wheat. On the other hand, the presence of S strains in sorghum may lead to crop contamination with high AF concentrations.

In conclusion, this study is the first report about the characterisation of *Aspergillus* section *Flavi* in Uruguay and shows that *A. flavus* type-L morphotype is the main fungal species infecting wheat and sorghum grains from the field to storage. The information generated in this work allows to contribute to the knowledge of the species in these crops mainly under silo bag storage conditions and to define appropriate strategies for the prevention and control of the contamination of grains with AFs and CPA. In addition, the existence of a non-toxigenic *A. flavus* population may be important in biological control of pre-harvest AFs contamination of crops through their application in the soil. A better understanding of genetic variability and fungal–fungal or host–fungal interactions within and between *Aspergillus* section *Flavi* populations must be done.

## Supplementary Material

Supplemental Material
