# Supplementary material for: Occurrence and toxigenic potential of Aspergillus section Flavi on wheat and sorghum silages in Uruguay
Source: Mycology. 2020 Apr 27;11(2):147–57. doi: 10.1080/21501203.2020.1752321 (PMC7448941; doi:10.1080/21501203.2020.1752321)
Supplement: Supplemental Material [file TMYC_A_1752321_SM9820.docx]

**Supplementary data**

Table S1. Diameter of growth in millimeters of the isolates obtained from wheat samples after seven days of incubation in different culture media and temperatures.

| Isolate | CYA 25°C | CYA 37°C | MEA 25°C | CZ 25°C | CY20S 25°C |
| --- | --- | --- | --- | --- | --- |
| 1 | 54 | 51 | 59 | 27 | 63 |
| 2 | 59 | 69 | 57 | 36 | 68 |
| 3 | 58 | 62 | 53 | 31 | 65 |
| 4 | 60 | 53 | 56 | 30 | 59 |
| 5 | 51 | 55 | 53 | 33 | 64 |
| 6 | 56 | 55 | 55 | 32 | 65 |
| 7 | 62 | 57 | 57 | 38 | 68 |
| 8 | 50 | 59 | 57 | 33 | 55 |
| 9 | 63 | 65 | 56 | 41 | 66 |
| 10 | 55 | 64 | 52 | 33 | 65 |
| 11 | 58 | 61 | 51 | 35 | 65 |
| 12 | 46 | 60 | 54 | 32 | 55 |
| 13 | 58 | 50 | 54 | 33 | 68 |
| 14 | 59 | 55 | 52 | 33 | 67 |
| 15 | 58 | 65 | 60 | 36 | 65 |
| 16 | 60 | 66 | 55 | 29 | 64 |
| 17 | 56 | 64 | 53 | 31 | 62 |
| 18 | 55 | 58 | 55 | 27 | 69 |
| 19 | 63 | 63 | 60 | 35 | 70 |
| 20 | 59 | 67 | 58 | 28 | 69 |
| 21 | 48 | 57 | 52 | 34 | 50 |
| 22 | 51 | 58 | 62 | 33 | 58 |
| 23 | 56 | 52 | 58 | 33 | 66 |
| 24 | 42 | 60 | 50 | 32 | 67 |
| 25 | 54 | 66 | 59 | 36 | 68 |
| 26 | 54 | 56 | 58 | 34 | 66 |
| 27 | 56 | 52 | 58 | 38 | 66 |
| 28 | 58 | 57 | 54 | 26 | 65 |
| 29 | 59 | 59 | 53 | 27 | 68 |
| 30 | 54 | 64 | 52 | 27 | 65 |
| 31 | 54 | 62 | 54 | 35 | 62 |
| 32 | 53 | 51 | 56 | 35 | 61 |
| 33 | 60 | 61 | 57 | 41 | 67 |
| 34 | 55 | 63 | 61 | 35 | 63 |
| 35 | 50 | 64 | 53 | 31 | 70 |
| 36 | 43 | 62 | 55 | 30 | 66 |
| 37 | 54 | 54 | 55 | 30 | 67 |
| 38 | 54 | 54 | 63 | 35 | 62 |
| 39 | 52 | 51 | 53 | 27 | 63 |
| 40 | 66 | 49 | 59 | 37 | 67 |

Table S2. Diameter of growth in millimeters of the isolates obtained from sorghum samples after seven days of incubation in different culture media and temperatures.

| Isolate | CYA 25°C | CYA 37°C | MEA 25°C | CZ 25°C | CY20S 25°C |
| --- | --- | --- | --- | --- | --- |
| 41 | 48 | 68 | 53 | 35 | 64 |
| 42 | 62 | 60 | 56 | 36 | 58 |
| 43 | 63 | 60 | 51 | 32 | 73 |
| 44 | 57 | 52 | 55 | 33 | 65 |
| 45 | 55 | 62 | 52 | 42 | 54 |
| 46 | 48 | 61 | 45 | 44 | 59 |
| 47 | 50 | 55 | 54 | 35 | 67 |
| 48 | 60 | 67 | 60 | 28 | 65 |
| 49 | 58 | 54 | 54 | 35 | 65 |
| 50 | 60 | 63 | 59 | 30 | 66 |
| 51 | 65 | 68 | 60 | 40 | 67 |
| 52 | 53 | 67 | 52 | 30 | 63 |
| 53 | 62 | 50 | 60 | 39 | 67 |
| 54 | 60 | 53 | 52 | 37 | 70 |
| 55 | 61 | 57 | 55 | 34 | 63 |
| 56 | 53 | 59 | 54 | 40 | 59 |
| 57 | 60 | 61 | 56 | 36 | 60 |
| 58 | 66 | 56 | 61 | 40 | 67 |
| 59 | 58 | 55 | 55 | 32 | 65 |
| 60 | 57 | 55 | 56 | 27 | 61 |
| 61 | 56 | 50 | 55 | 33 | 62 |
| 62 | 56 | 55 | 53 | 30 | 62 |
| 63 | 58 | 61 | 54 | 34 | 66 |
| 64 | 55 | 56 | 51 | 34 | 60 |
| 65 | 64 | 65 | 58 | 33 | 63 |
| 66 | 58 | 62 | 53 | 33 | 66 |
| 67 | 56 | 60 | 54 | 34 | 66 |
| 68 | 46 | 61 | 45 | 44 | 59 |
| 69 | 46 | 64 | 50 | 36 | 55 |
| 70 | 50 | 53 | 53 | 32 | 55 |
| 71 | 59 | 55 | 50 | 31 | 56 |
| 72 | 64 | 67 | 54 | 30 | 66 |
| 73 | 57 | 52 | 63 | 31 | 68 |
| 74 | 59 | 55 | 50 | 31 | 56 |
| 75 | 48 | 66 | 40 | 42 | 59 |
| 76 | 55 | 65 | 53 | 37 | 63 |
| 77 | 59 | 57 | 57 | 30 | 56 |
| 78 | 56 | 67 | 55 | 33 | 65 |
| 79 | 55 | 64 | 57 | 36 | 68 |
| 80 | 48 | 67 | 49 | 35 | 65 |
